# Pharmacological Investigation of Fluoro-Gold Entry into Spinal Neurons

**DOI:** 10.1371/journal.pone.0131430

**Published:** 2015-06-23

**Authors:** Melanie Falgairolle, Michael J. O’Donovan

**Affiliations:** Section on Developmental Neurobiology, National Institute of Neurological Disorders and Stroke, National Institutes of Health, Bethesda, Maryland, United States of America; IIBB/CSIC/IDIBAPS, SPAIN

## Abstract

The fluorescent tracer Fluoro-Gold has been widely used to label neurons retrogradely. Here we show that Fluoro-Gold can also enter neurons through AMPA receptor endocytosis. We found that a 30 minute application of Fluoro-Gold to the isolated spinal cord labeled neurons under control conditions and in the presence of glutamatergic agonists including NMDA and AMPA. The labeling was abolished or greatly reduced by glutamatergic antagonists and the endocytic inhibitors Dynasore and dynamin inhibitory peptide. Whole cell recordings from spinal neurons exposed to extracellular AMPA revealed large inward currents that spontaneously decayed in the presence of the agonist but were maintained when a dynamin inhibitory peptide was included in the electrode. These findings suggest that Fluoro-Gold enters spinal neurons through AMPA-mediated receptor internalization. Drugs used to induce locomotor-like activity in the spinal cord also increased and decreased Fluoro-Gold labeling in a drug and lamina specific manner, indicating that AMPAR endocytosis is altered in the presence of the locomotor cocktail. Our findings suggest that endocytosis of Fluoro-Gold could potentially complicate the interpretation of experiments in which the tracer is used to label neurons retrogradely. Moreover, they also demonstrate that many drugs, including the locomotor cocktail, can modulate the number and/or the composition of AMPA receptors on spinal neurons and thereby affect network excitability.

## Introduction

AMPA (α-Amino-3-hydroxy-5-methyl-4-isoxazolepropionic acid) receptors (AMPARs) mediate fast synaptic transmission in the mammalian central nervous system. Their number is actively regulated by membrane trafficking and this process underlies many forms of synaptic plasticity [1–6]. In the rodent spinal cord, glutamatergic transmission is integral to the operation of the central pattern generator [7–10]. For example, glutamate receptors are involved in the control of locomotor speed [11] and activation of AMPARs is required to elicit a high-frequency locomotor-like rhythm [10]. In addition, AMPARs are critical in pain pathways and have been shown to contribute to low-threshold afferent drive into the dorsal horn [12], and are also involved in activity-dependent changes in the synaptic processing of nociceptive inputs [13]. Moreover, Park et al. [14] have shown that persistent inflammation can cause AMPARs to internalize and other evidence suggests that spinal cord injury and excitotoxicity can alter AMPA receptor trafficking [15].

FG has been widely used to label neurons retrogradely [16–18]. In contact with cut axons, the dye is incorporated intracellularly and transported retrogradely to the soma probably within endosomal organelles [19]. Here, we show that bath-application of FG leads to neuronal uptake in a non-retrograde manner. We demonstrate that the number of FG-labeled neurons increased or decreased with activation or blockade of ionotropic GluRs (NMDAR, AMPAR, and KAR) respectively and was particularly sensitive to AMPAR agonists. Dynasore and dynamin inhibitory peptide, inhibitors of endocytic pathways, reduced FG labeling by AMPA administration suggesting that the uptake mechanism involved AMPAR-mediated endocytosis of bath-applied FG.

Little is known, however, about the role of AMPA receptor trafficking in the operation of spinal motor networks including the locomotor central pattern generator (CPG). This is important because many of the drugs that are used to activate the locomotor CPG can alter AMPA receptor trafficking acutely. For example, NMDA is known to trigger endocytosis of AMPARs leading to long term depression in the hippocampus [20]. In the prefrontal cortex, both dopamine [21] and serotonin [22] can lead to AMPAR internalization. Bath application of NMDA, serotonin and dopamine are commonly used to trigger locomotor-like activity in the neonatal rodent cord but little is known about how these drugs affect AMPAR trafficking. In this paper, we show that Fluoro-Gold can enter spinal neurons through AMPA-mediated AMPAR endocytosis. We also show that the drugs used to activate locomotion in the neonatal spinal cord [23] all had effects on FG labeling suggesting that they alter AMPAR trafficking and therefore could modify the properties of locomotor networks. Some of this work has been published in abstract form [24].

## Material and Methods

### Mice

All experiments were carried out in compliance with the National Institutes of Neurological Disorders and Stroke Animal Care and Use Committee (Animal Protocol Number 1267–09 and 1267–12).

### Reagents

Dyes and drugs were purchased from the following suppliers: (RS)-AMPA hydrobromide (AMPA), Kainate (KA), GYKI 52466 hydrochloride (GYKI), DL-*threo*-β-Benzyloxyaspartic acid (TBOA), NBQX disodium salt (NBQX), DL-AP5 sodium salt (APV), dynamin inhibitory peptide (QVPSRPNRAP), dynamin inhibitory peptide myristoylated (scrambled), dynamin inhibitory peptide myristoylated, cyclothiazide, mecamylamine, dhβe, and Dynasore (Tocris, Ellisville, MO, USA); N-Methyl-D-Aspartate (NMDA), tetrodotoxyn (TTX), Strychnine, Bicuculline and L-glutamate, atropine (Sigma, St Louis, MO); Fluoro-Gold (Fluorochrome, Inc., Denver, CO, USA); Lucifer Yellow (LY), Acridine Orange 10-Nonyl (AO), 6-methoxy-*N*-ethylquinolinium iodide (MEQ), and Florescien-5-isothiocyan (FITC) were from Invitrogen. Dynasore was dissolved in DMSO. When bath applied to the cord, the concentration of DMSO was less than 0.1%. All other drugs were dissolved in artificial cerebrospinal fluid (aCSF).

### Dissection

Experiments were performed on three-day-old Swiss Webster mice (Taconic and Jackson laboratory and Charles River). The mice were decapitated and eviscerated then placed in a dissecting chamber and continuously perfused with aCSF (concentrations in mM: 128.35 NaCl, 4 KCl, 1.5 CaCl_2_.H2O, 1 MgSO_4_.7H_2_O, 0.58 NaH_2_PO_4_.H2O, 21 NaHCO_3_, 30 D-glucose) bubbled with 95% O_2_ and 5% CO_2_. After a ventral laminectomy, the cord was isolated together with the attached roots and ganglia and maintained at room temperature.

### Fluoro-Gold Labeling

For Fluoro-Gold labeling, the cord was transferred to a tube containing 2.5 or 5ml aCSF and constantly bubbled with 95% O_2_-5% CO_2_. Under control conditions (no drugs or stimulation) Fluoro-Gold (200 μM) was added to the aCSF for 30 minutes. To test the effect of drugs on the loading of Fluoro-Gold into cells, the drugs were applied for 10 min, followed by co-application of the drug and Fluoro-Gold (200 μM) for a further 30 minutes. Exposure of the cord for longer (60 min.) with a higher Fluoro-Gold concentration (500 μM) did not improve the loading, therefore we routinely used Fluoro-Gold at a concentration of 200 μM. Each condition was reproduced at least three times on mice from different litters. The cords were then fixed in 4% paraformaldehyde at room temperature for no less than 4 hours. They were then cut to retain only two segments (spanning L4–L6) and finally washed in 0.01M phosphate buffered saline (PBS). The segments were then embedded in warm 5% Agar and transverse sections (60 μm) were cut on a vibratome (Leica, V1000). The first 10 to 15 intact sections were mounted on slides and cover-slipped with a solution made of Glycerol and PBS (3:7). Images were acquired using a LSM510 Carl Zeiss confocal microscope with 10x objective (NINDS, light facility).

### Statistical comparison of the spatial distribution of Fluoro-Gold-labeled cells under different experimental conditions

**Fig 1:**
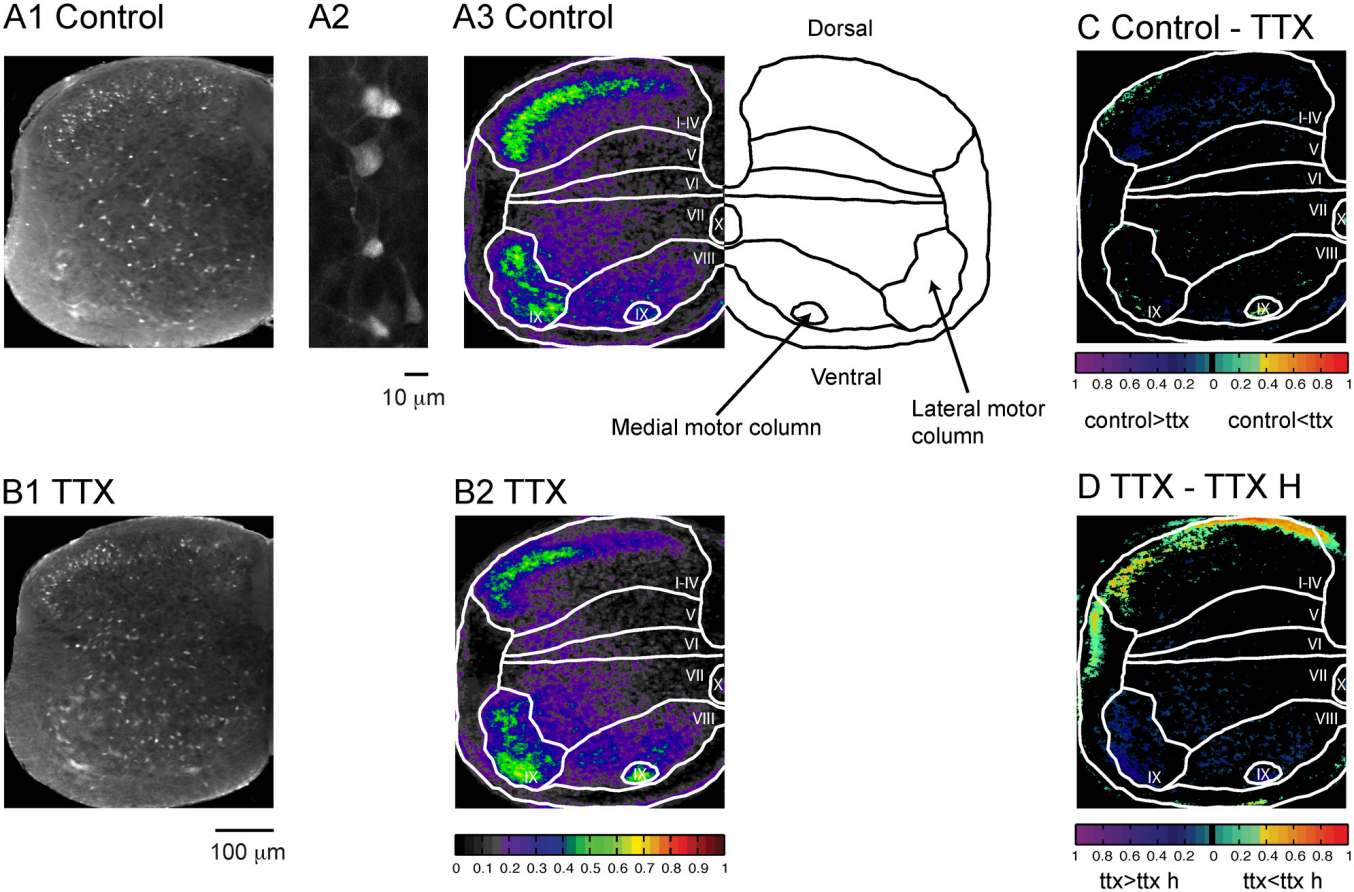
Fluoro-Gold uptake under control conditions is not retrograde or action potential-dependent. (A1) Image of a hemicord section, from a cord in which Fluoro-Gold was bath-applied for 30 minutes, showing labeling of motoneurons and interneurons. (A2) Higher magnification of labeled interneurons. (A3) Map showing the probability of finding a labeled cell at a particular pixel. Note that in this and other figures only a hemicord is shown. The schematic in this panel outlines the motor columns and provides dorsal and ventral orientation. (B1) Labeling pattern recorded in the presence of TTX. (B2) Probability map for the TTX condition. The color map showing the probability of labeling for A3 and B2 is shown beneath the panel in B2. (C) Difference map comparing the probability of labeling under control conditions and in the presence of TTX. The color map beneath the figure shows the color coding for pixels that differed statistically (p <0.05) between control and TTX labeling. (D) Difference map comparing the probability of labeling in the presence of TTX for 10 minutes and for 1 hour before adding FG. The color map beneath the figure shows the coding for pixels that differed statistically (p <0.05).

We will provide a full description of the bootstrap method for quantifying and statistically comparing labeling patterns in the spinal cord in a forthcoming publication. Briefly, 10 different sections from each cord were scanned and the resulting images were cropped to one side of the cord and then rotated and/or flipped to allow them to be subsequently aligned. All objects in the image that were not part of the spinal cord *per se* (ventral and dorsal roots, agar) were removed digitally. Images were then compressed to 640 X 480 format so that they could be combined and averaged. The images were then processed with ImageJ [25] to enhance the contrast by removing the background and doubling the intensity of each pixel. We subsequently applied edge detection and saved both images. The generation of the final binary image (1 for labeling, 0 for no labeling) was performed using custom written Matlab code. The two images generated using ImageJ were analyzed separately. We first filled the structures that were detected in the edge image. Then areas of interest were identified in both images using an intensity threshold and were combined. Only round structures greater than 8 pixels in diameter were kept. Finally, the resulting image was converted into a binary image. Typically 10 images from three cords (30 images in total) were combined into a 640x480x30 matrix. Each voxel in the matrix comprised 30 entries of either a 1 or a 0. These values were averaged to generate a pseudo-colored image where each pixel represented the probability of finding a cell at that location (see Fig 1A3). To identify regional variations in labeling we superimposed a map of the Rexed laminae (adapted from [26]) over the pseudo-colored image. The probability of finding a labeled pixel in each lamina or group of laminae was determined by averaging all of the pixel probability values within the region (see Tables 1–3). To compare two maps generated under different experimental conditions (e.g. control vs drug; drug vs drug) we used a bootstrap method to test whether two maps were different statistically. Each sample (voxel) was a vector comprised of at least of 30 values. To compare the vectors generated from two different maps, we averaged each vector and then subtracted the averaged values. The bootstrap method establishes the likelihood that this difference could have occurred by chance. The method concatenates the 2 vectors into a new vector with 60 values, and then generates two new vectors of 30 values each by randomly selecting values from the concatenated vector. This process is repeated 1000 times and the distribution of the averaged, subtracted values generated by these two sets of 1000 random vectors is used to establish the likelihood that the actual difference between the original vectors could have arisen by chance.

**Table 1:** Effects of glutamatergic agents and TTX on Fluoro-Gold uptake in different laminae of the spinal cord.

Conditions	LAMINAE						
	I-IV	V	VI	VII	VIII	IX (lateral)	IX (medial)
Control	0.21	0.09	0.08	0.13	0.19	0.32	0.28
TTX	0.16 (-8.2)	0.07 (-1.5)	0.07 (-0.5)	0.13 (-0.4)	0.19 (-1.2)	0.33 (-0.0)	0.36 (13.0)
TTX-1hour	0.18 (12.3)	0.019 (-11.1)	0.014 (-11.5)	0.028 (-10.2)	0.044 (-11.9)	0.10 (-32.6)	0.09 (-39.3)
APV	0.14 (-14.0)	0.05 (-4.5)	0.05 (-3.0)	0.10 (-3.1)	0.11 (-10.2)	0.25 (-7.3)	0.24 (-1.9)
NBQX	0.13 (-16.6)	0.06 (-3.8)	0.05 (-1.3)	0.07 (-5.7)	0.10 (-12.5)	0.23 (-9.6)	0.20 (-5.6)
APV + NBQX	0.06 (-37.0)	0.06 (-3.8)	0.02 (-6.5)	0.04 (-14.6)	0.08 (-20.4)	0.16 (-24.1)	0.18 (-6.3)
AMPA	0.62 (78.2)	0.46 (95.1)	0.41 (92.7)	0.48 (89.5)	0.6 (90.7)	0.75 (86.7)	0.82 (98.6)
NMDA	0.35 (25.4)	0.08 (-2.21)	0.1 (0.9)	0.15 (0.9)	0.18 (-2.8)	0.40 (17.0)	0.40 (18.3)
KA	0.65 (69.0)	0.16 (14.1)	0.07 (1.5)	0.17 (5.1)	0.42 (38.0)	0.73 (68.3)	0.78 (94.2)
TBOA	0.22 (12.9)	0.06 (1.4)	0.04 (1.8)	0.07 (5.1)	0.18 (38.0)	0.26 (68.3)	0.35 (9.9)
Glutamate	0.31 (6.6)	0.08 (-1.0)	0.05 (-0.4)	0.08 (-2.4)	0.13 (0.1)	0.28 (-3.9)	0.25 (3.2)

The first number is the probability of labeling/pixel and the number in parentheses is the percentage of pixels in the lamina that were greater or lesser (negative values) than control labeling at p <0.05. See text for details.

**Table 2:** Effects of glutamatergic antagonists and blockers of endocytosis on labeling.

Conditions	LAMINAE						
	I-IV	V	VI	VII	VIII	IX (lateral)	IX (medial)
NMDA + NBQX	0.17	0.02	0.02	0.04	0.05	0.12	0.11
AMPA + APV	0.58	0.28	0.09	0.20	0.46	0.54	0.67
Kainate + NBQX	0.25	0.04	0.02	0.05	0.08	0.21	0.34
NMDA + NBQX vs NBQX	3.02	-2.33	-1.96	-2.45	-5.26	-18.68	-6.85
AMPA + APV vs APV	92.95	53.86	9.36	31.30	86.47	71.52	98.00
KA + NBQX vs NBQX	21.01	-3.27	-8.38	-1.63	2.22	-2.69	12.98
Sucrose	0.10	0.02	0.01	0.03	0.04	0.15	0.10
Sucrose + AMPA	0.58	0.28	0.11	0.24	0.45	0.63	0.61
Sucrose + AMPA vs AMPA	-25.61	-49.19	-77.76	-57.75	-30.19	-46.38	-36.63
Dynasore	0.00	0.00	0.00	0.00	0.00	0.00	0.00
Dynasore + AMPA	0.23	0.03	0.01	0.03	0.10	0.25	0.33
Dynasore + AMPA vs AMPA	-76.57	-99.78	-98.65	-98.38	-96.71	-91.25	-96.46

For the six first entries, the top three show the labeling probability/pixel for the different drugs. The last three show the percentage of pixels that differ between the two mentioned conditions (p <0.05). After that, the top two entries show the labeling probability/pixel for the different drugs whereas the last one shows the percentage of pixels that differ between the two conditions (p <0.05).

**Table 3:** The Effects of locomotor drugs on Fluoro-Gold uptake in different laminae of the spinal cord.

Conditions	LAMINAE						
	I-IV	V	VI	VII	VIII	IX (lateral)	IX (medial)
Control	0.21	0.09	0.08	0.13	0.19	0.32	0.28
Dopamine	0.28 (19.2)	0.05 (-2.5)	0.05 (-0.9)	0.07 (-4.5)	0.12 (-8.6)	0.29 (-2.3)	0.39 (14.1)
Serotonin	0.35 (28.4)	0.04 (-7.3)	0.03 (-5.2)	0.04 (-17.8)	0.05 (-35.2)	0.14 (-43.2)	0.08 (-51.2)
Dopamine + Serotonin	0.32 (19.22)	0.02 (-10.9)	0.02 (-6.3)	0.02 (-21.7)	0.05 (-31.8)	0.15 (-37.9)	0.19 (-7.6)
NMDA vs Control	0.35 (25.4)	0.08 (-2.21)	0.1 (0.9)	0.15 (0.9)	0.18 (-2.8)	0.40 (17.0)	0.40 (18.3)
TTX	0.16	0.07	0.07	0.13	0.19	0.33	0.36
TTX-Dopamine	0.43 (56.1)	0.1 (3.3)	0.08 (1.6)	0.14 (2.9)	0.22 (4.1)	0.42 (7.6)	0.37 (-3.1)
TTX-Serotonin	0.42 (50.2)	0.04 (-1.2)	0.03 (-3.9)	0.05 (-12.7)	0.08 (-16.1)	0.15 (-34.6)	0.11 (-55)
TTX-NMDA	0.31 (33.6)	0.04 (-1.6)	0.07 (2)	0.10 (-0.3)	0.20 (0.9)	0.22 (-18.2)	0.25 (-22.7)
TTX-AMPA	0.68 (96.6)	0.49 (99)	0.47 (99.2)	0.50 (99.3)	0.58 (93.5	0.67 (74)	0.69 (62.2)

The first number is the probability of labeling/pixel and the number in parentheses is the percentage of pixels in the lamina that were greater or lesser (negative values) than control labeling at p <0.05. See text for details. The effects of NMDA have also been reproduced from Table 1 because this drug is frequently included in the locomotor cocktail.

We considered two populations being different when the p-value was less than 0.05. This allowed us to build a pseudo-colored map (difference map) showing only the differences in the dye uptake between those two conditions that were statistically different (P < 0.05; see Fig 1C). To quantify the changes per lamina, we calculated the percentage of pixels that were statistically increased or decreased (P < 0.05) between laminae under the experimental condition versus its control. Because pixel probabilities both increased and decreased for each comparison we subtracted the negative pixel probability from the positive pixel probability to provide an estimate of the overall change for the laminar comparisons. Tables 1 to 3 report this value and the two conditions that were used to calculate it.

### Whole cell recordings

Microelectrodes were pulled from borosilicate capillaries with a microelectrode puller (model p-80; Sutter Instruments). Pipettes (7.8–12.6MΩ) were filled with intracellular solution (NaCl 10 mM; K-Gluconate 130mM; MgCl_2_ 1mM; HEPES 10 mM; Na_2_GTP 0.2 mM; MgATP 1 mM; EGTA 11 mM; CaCl_2_ 0.1 mM; pH adjusted to 7.2–7.3 with KOH). To block endocytosis, Dynamin inhibitory peptide (100 μm) was added to the intracellular solution. Experiments were performed in whole-cell patch clamp configuration using either a Multiclamp 700B amplifier or a Multiclamp 700A. Series resistance was not compensated. Recordings were acquired at 10kHz and filtered at 3kHz.

## Results

### Fluoro-Gold labeling under control conditions

FG is the di-(hydroxyethanesulfonate) salt of hydroxystilbamidine, a water soluble molecule (532.6 Da) that dissociates into acidic hydroxyethanesulfornate and basic hydroxystilbamidine ions. Hydroxystilbamidine (MW 280.3 Da) is believed to be membrane-impermeant [19], and is the active component of the fluorescent dye [27]. We first examined the spatial pattern of Fluoro-Gold labeling in the L4–L6 segments of the lumbar spinal cord under control conditions (no drugs added, no stimulation). The dye was applied for 30 minutes and the dorsal and ventral roots were kept as long as possible to minimize the possibility of the anterograde and retrograde labeling known to occur with Fluoro-Gold [19, 28]. Under these conditions, cells were labeled throughout the grey matter (Fig 1A1 Control). To visualize the likelihood of finding a labeled pixel at a particular location, we generated a map of the labeling probability (see material and methods). This map revealed that the lateral motor nucleus and lamina I-IV showed the highest labeling probability with many individual pixels having probability values > 0.5 (Fig 1A3, Control). Within laminae I-IV, labeling appeared to be predominantly in lamina II, although we did not attempt to subdivide laminae I-IV because their boundaries are hard to define in the neonatal cord. The lowest labeling probabilities were found in laminae V and VI. To provide a measure of the labeling within the laminae, we averaged all of the pixel probability values—including zeros—within individual laminae to generate a probability/pixel for that lamina. The average probability/pixel was highest in the lateral and medial motor columns (0.32 and 0.28 respectively) and was lowest in laminae V and VI (0.09 and 0.08 respectively).

### Short term dye uptake under control conditions is largely independent of neuronal firing

We then established whether or not the labeling pattern generated under control conditions was activity-dependent. For this purpose, we bath-applied TTX (5 μM) to the cord 10 minutes before adding Fluoro-Gold for a further 30 min. (Fig 1B). To compare the loading between control cords and those labeled in the presence of TTX, we generated a difference map (Fig 1C) showing those pixels that differed statistically between the two conditions (P < 0.05; see methods). We found that the labeling distribution was similar between the two conditions (Fig 1A3 Control, 1B2 TTX) although the dorsal interneurons from lamina I-IV were less likely to be labeled (Fig 1C) and to a lesser extent those in the intermediate zone (lamina VII, Fig 1C). The percentage of pixels that were lower than control (p < 0.05) in the presence of TTX (Table 1) ranged from -0.4% (lamina VI) to -8.2% (lamina I-IV). Only the medial motoneuron pool showed an increase of labeling (lamina IX medial, 13%) in the presence of TTX. However, when TTX was incubated for an hour before adding FG there was a decrease (Table 1) of labeling in all laminae except for lamina IV when compared to TTX application for 10 minutes (Fig 1D). These results confirm a previous study [29] showing that application of TTX reduced the rate of AMPAR internalization after an hour of application, and indicate that the influence of TTX on the uptake of Fluoro-Gold is time dependent and activity-dependent.

### Dye uptake is glutamate receptor-dependent

**Fig 2:**
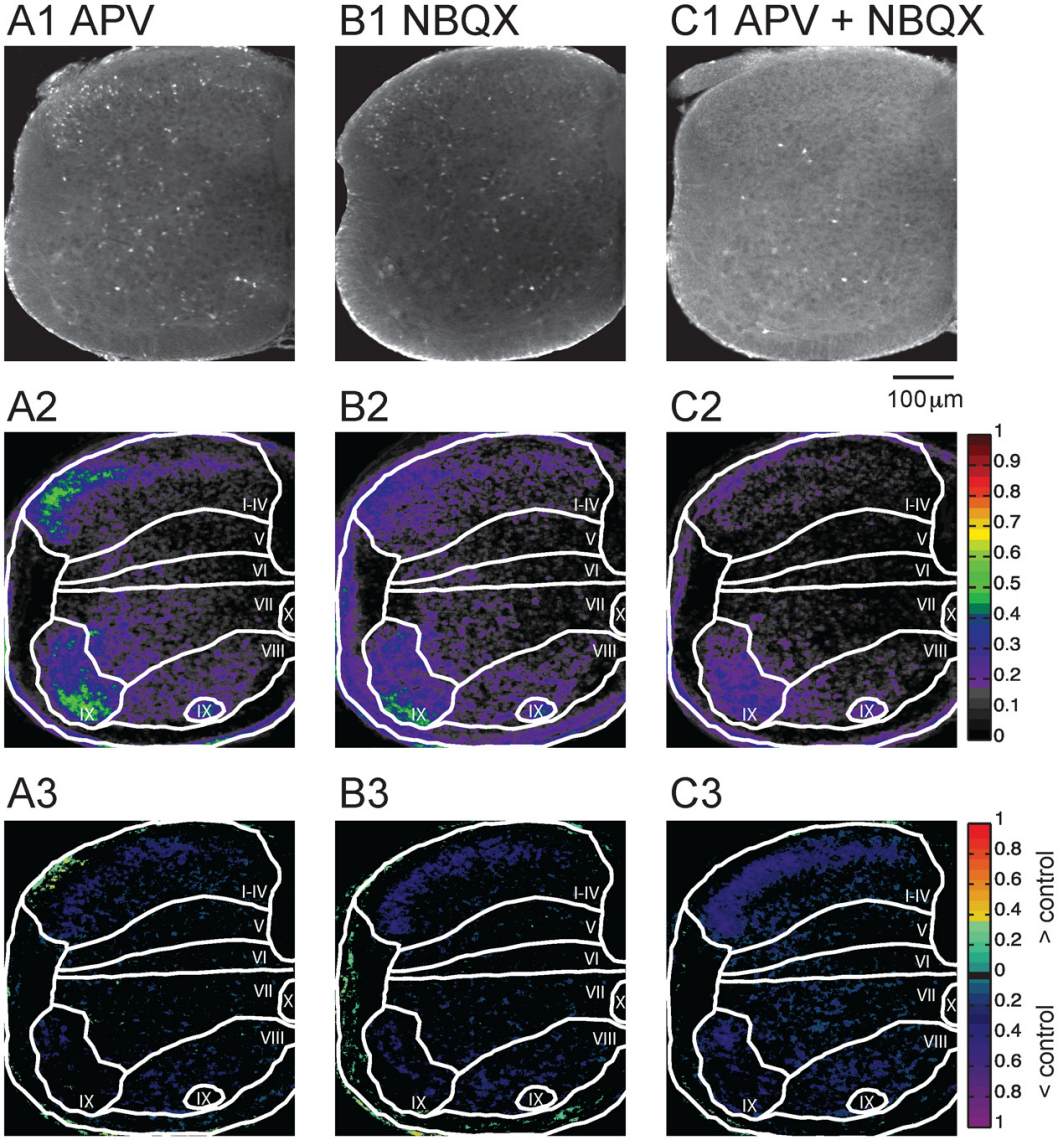
Glutamate antagonists reduce Fluoro-Gold labeling. (A1 –A3) Application of APV (50 μM) together with Fluoro-Gold. (A1) Image of labeling pattern, (A2) Probability map of labeling distribution, (A3) Difference map comparing the APV map to the control map. (B1–B3) Application of 20 μM of NBQX. (B1) Image of labeling pattern, (B2) Probability map of labeling distribution, (B3) Difference map comparing NBQX map to control map. (C) Application of APV and NBQX simultaneously. (C1) Image of labeling pattern, (C2) Probability map of labeling distribution, (A3) Difference map comparing NBQX+APV map to control map. The colormap to the right of panels A2 –C2 indicates the probability of labeling. The colormap to the right of A3 –C3 indicates labeling greater than control (green to red) or less than control (purple to blue) at P < 0.05.

To establish if Fluoro-Gold labeling depended on glutamatergic neurotransmission we applied NMDAR and AMPAR antagonists (APV 50μm and NBQX 20μm respectively) to the cord 10 minutes before adding the dye (Fig 2). In the presence of APV (Fig 2A1–2A3), labeling was observed in all laminae (Fig 2A1 and 2A2). Compared to control (no drug), however, there was a decrease in the probability of dye uptake in all areas (Fig 2A3), particularly in lamina I-IV (probability/pixel: control 0.21; APV 0.14) and the lateral motoneuron column (probability/pixel: control 0.32; APV 0.25). We then examined the effects of the AMPAR antagonist NBQX and found that the spatial distribution of labeling was similar to control but reduced throughout (Fig 2B1–2B3), particularly in laminae I-IV (probability/pixel: control 0.21; NBQX 0.13) and laminae IX (probability/pixel: control 0.32; NBQX 0.23). Thus, similar to NMDAR blockade, AMPAR blockade led to reduced labeling. Collectively, these results suggest that the control labeling pattern was mediated by extracellular glutamate.

When we applied both NBQX and APV (Fig 2C1–2C3) most of the labeling disappeared in all laminae. For example, labeling in the lateral motor column (IX) fell from a probability/pixel of 0.32 to 0.16. Even in laminae (V–VIII) where the labeling was weakest under control conditions, the averaged probability declined in the presence of APV and NBQX. The effects of these drugs on the probability/pixel and on the percentage of pixels that differed between the drug and control are shown in Table 1. In summary, under all three conditions, there was a reduction of the labeling probability compared to the control condition; neither APV nor NBQX alone was able to block completely cellular labeling. However, when both drugs were co-applied most of the dye uptake was abolished.

### Fluoro-Gold uptake is enhanced by application of glutamate agonists

**Fig 3:**
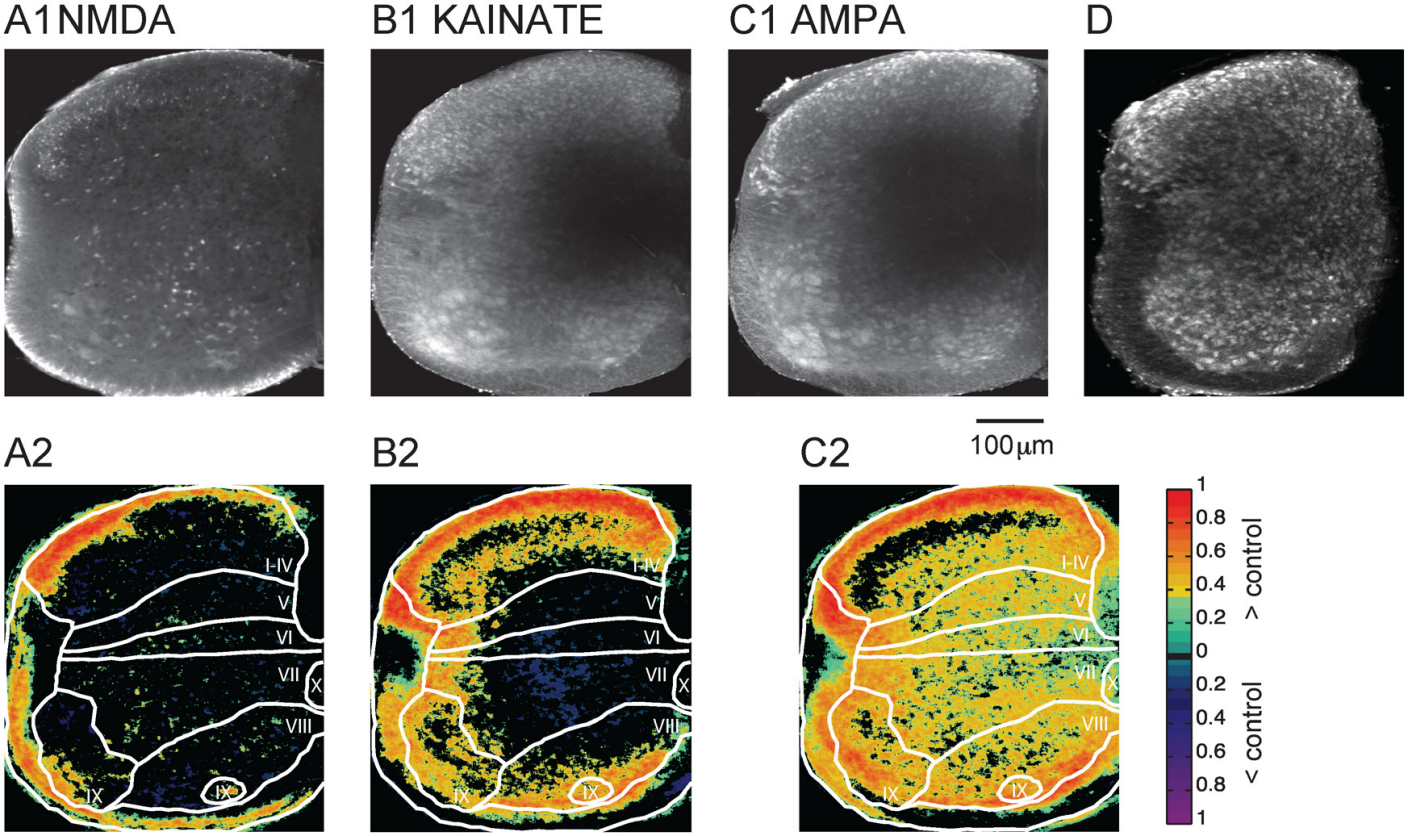
Glutamate agonists enhanced the uptake of Fluoro-Gold. (A1) Application of NMDA (20 μM) results in increased labeling compared to control. (A2) Difference map comparing NMDA labeling to control. (B1) Application of Kainate (10 μM) led to a significant increase in cellular labeling. (B2) Difference map of Kainate labeling compared to the control condition. (C1) Application of AMPA (5 μM) led to a similar increase in labeling compared to Kainate application (C2) Difference map comparing AMPA labeling to control. The difference maps only display pixels that differed from the control at P<0.05. The colormap to the right of A2, B2, and C3 shows the color coding for pixels that differed statistically (p<0.05) from control. D) Application of AMPA to a hemicord eliminated the central region of low labeling.

The effects of glutamate antagonists on Fluoro-Gold labeling suggested that glutamatergic transmission was involved in mediating Fluoro-Gold uptake into neurons. To confirm this idea, we applied Fluoro-Gold together with the following glutamatergic agonists: NMDA (20 μM), kainate (10 μM), or AMPA (5 μM). Fig 3 shows examples of the cellular labeling patterns resulting from these experiments. As previously described, all agonists were pre-applied and Fluoro-Gold was added 10 minutes later.

Application of NMDA led to increased labeling particularly in laminae I-IV where it preserved the original control pattern but increased the probability of labeling (Fig 3A and Table 1). For most of the remaining regions, the distribution was either similar to control or slightly elevated. The probability/pixel in the presence of NMDA rose from a control level of 0.32 and 0.28 in the lateral and medial motor columns respectively to 0.4 in both regions. The corresponding increases in the percentage of labeled pixels were 17% and 18.3% in the lateral and medial columns. The probability of labeling was lower than control in laminae V and VIII. The edges of the cord were also often very brightly labeled and therefore they appeared to have a higher uptake probability. The meninges were usually labeled in all conditions, and especially in presence of NMDA. When either kainate or AMPA was applied to the cord, almost all neurons were labeled except for the central part of the cord (Fig 3B and 3C) and this pattern differed significantly from the control cords. Processes were also brightly labeled and hence the white matter appeared as labeled.

Because the maps were generated to detect labeled pixels independently of their intensity, they do not reflect the decrease in the intensity of labeling from the edges to the center of the cord. All laminae exhibited an increase in the probability of labeling and in the percentage of labeled pixels. In the presence of AMPA, the percentage of labeled pixels increased by 78.2–98.6% in the different laminae with the largest increases of 95.1 and 98.6% in lamina V and the medial motor nuclei respectively. However in the lamina I-IV and the lateral motoneuron pool, the differences between control and AMPA were not significantly different (hence the black zones in the map, Fig 3C3). The weaker labeling in the medial portion of the cord is probably due to limited diffusion of the dye into that region because it was much less evident when a hemicord was labeled (Fig 3C2).

**Fig 4:**
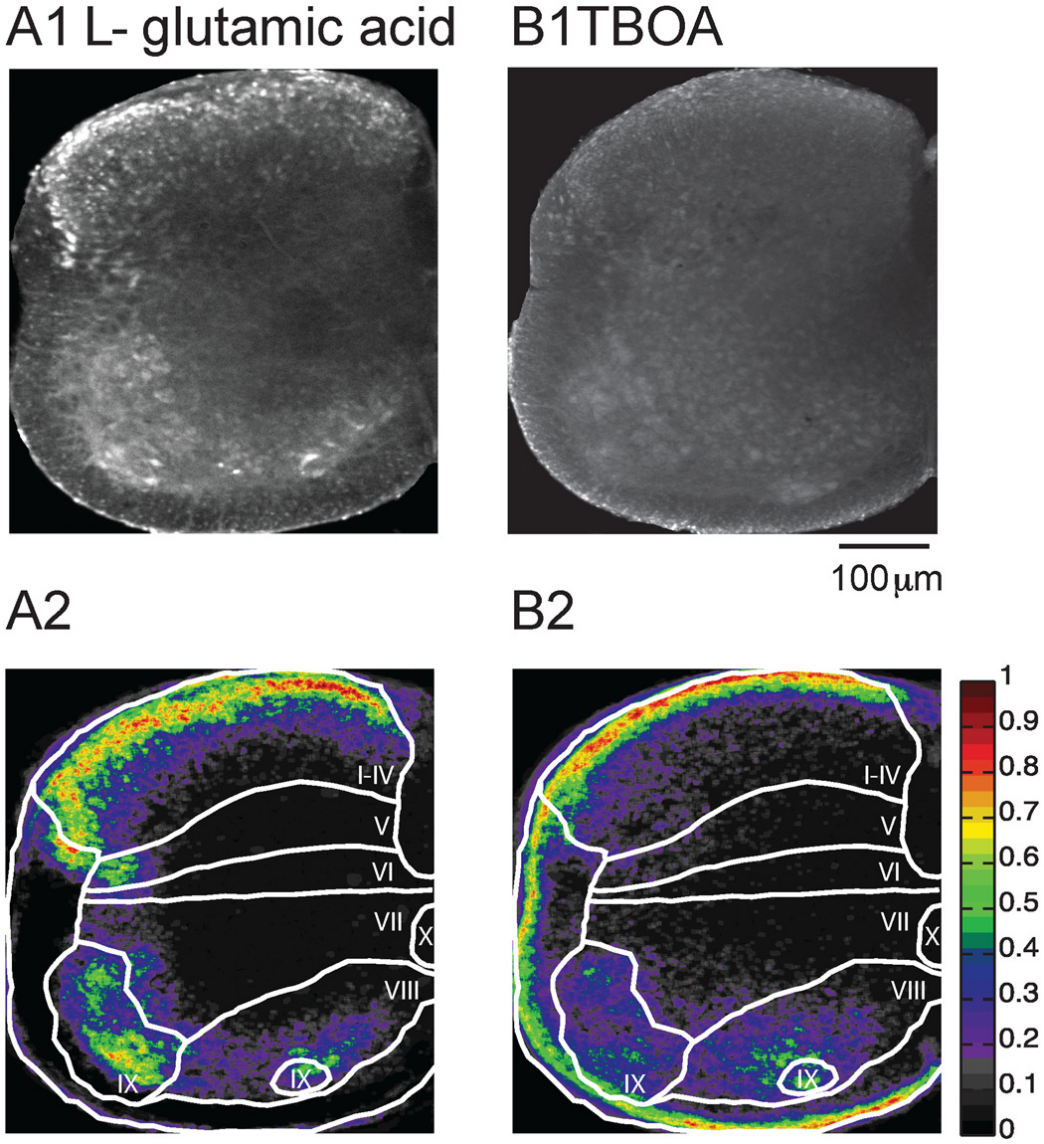
L-glutamic acid as well as TBOA induced uptake of Fluoro-Gold. (A1) Application of 5mM L-glutamic acid resulted in widespread labeling but not as extensive as that obtained with AMPA or Kainate administration. (A2) Probability map for L-glutamate-induced uptake. (B) Application of TBOA (50 μM) also led to neuronal uptake. (B2) Probability map for TBOA. The drug was applied for 10 minutes and a further 30 min in the presence of Fluoro-Gold. The color map to the right of A2 and B2 shows the probability of labeling.

In the next set of experiments, we examined the effects of the endogenous excitatory neurotransmitter L-glutamate on the uptake of Fluoro-Gold. Application of glutamate (5mM) created an uptake pattern that was similar but less pronounced compared to AMPA or Kainate application (Fig 4A1 and 4A2). We also examined influence of endogenous glutamate release by applying TBOA (50 μM), a glutamate uptake blocker. Consistent with the presence of endogenous glutamate in the hemicord, we found labeling in the gray matter in the presence of the uptake blocker. For both drugs the meninges were brighter than the rest of the cord (Fig 4B). However, some labeling could be still observed in gray matter.

### Fluoro-Gold uptake depends primarily on AMPA receptor activation

**Fig 5:**
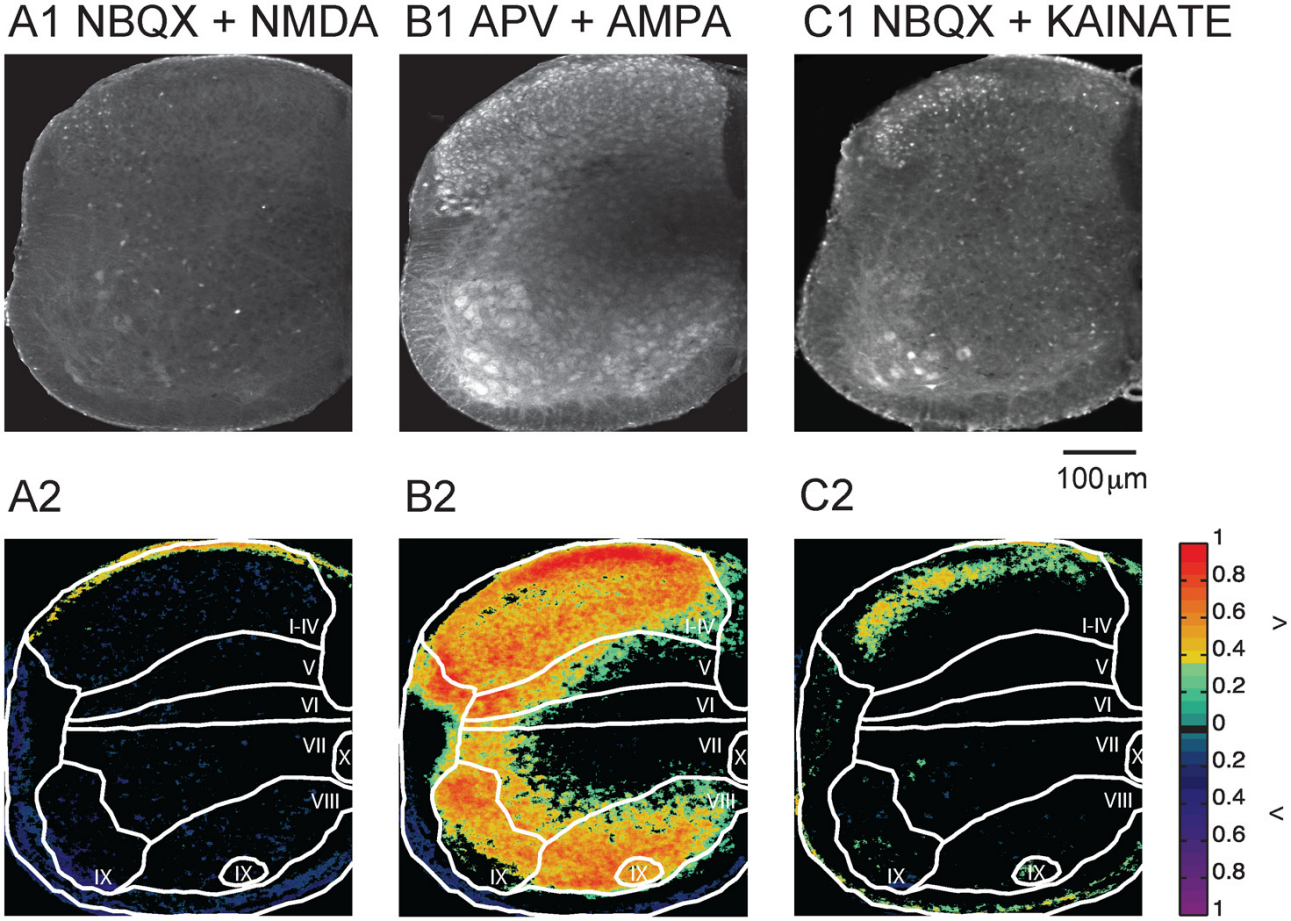
AMPA receptors were responsible for most of the glutamatergic dye uptake. (A1) Application of NMDA (20 μM) together with NBQX (20 μM) led to reduced labeling compared to NBQX alone. (A2) Difference map obtained by subtracting the probability map for the NMDA and NBQX from the probability map for NBQX alone. (B1) Application of both AMPA (5 μM) and APV (50 μM) resulted in a labeling pattern similar to that observed with the application of AMPA alone. (B2) Difference map obtained by subtracting the probability map for the AMPA + APV from the probability map for APV alone. (C1) Application of both Kainate (10 μM) and NBQX (20 μM) abolished most of the cellular labeling. (C2) Difference map obtained by subtracting the probability map for the Kainate and GYKI from the probability map for NBQX alone. The colormap to the right of A2, B2, and C2 shows the color coding for pixels that differed statistically (p<0.05) from control.

We have shown that glutamate receptors are involved in the labeling of the cord with Fluoro-Gold, and that the activation of each receptor type can enhance the uptake probability. To test the contribution of each receptor subtype to the uptake pattern we investigated the effects of agonist application in the presence of glutamate receptor antagonists. When uptake was examined in the presence of NMDA and the AMPA and Kainate receptor antagonist NBQX, most of the cellular labeling disappeared (Fig 5A).

For example, the probability/pixel for laminae I-IV fell from 0.21 in the control (no drugs, Table 1) to 0.17 in the presence of NMDA and NBQX. In lamina IX (lateral motor column) the corresponding values were 0.32 (control) to 0.12 (drugs). When compared to NBQX application all laminae exhibited a lower labeling probability/pixel with the exception of laminae I-IV, (Fig 5A2; Table 2).

When AMPA was co-applied with the NMDAR antagonist APV (50μM), the labeling pattern was similar to that produced by application of AMPA alone (Fig 5B; Table 2). A strong probability of labeling could be seen in laminae I-IV, VIII and IX (I-IV = 0.58, VIII = 0.46, IX lateral = 0.54 and IX medial = 0.67). When compared to the application of APV alone to the cord, large differences were observed (Fig 5B2, Table 2). Finally, when we added kainate and NBQX most of the labeling was abolished (Fig 5C1, Table 2). While there was an increase in the percentage of pixels labeled in lamina I-IV (+21.01%) and medial lamina IX (+12.98%) compared to NBQX alone, in the other laminae the labeling was similar to NBQX (Fig 5C2, Table 2). These results suggest that the uptake of Fluoro-Gold in the presence of each of these glutamatergic agonists is mediated primarily through activation of AMPA receptors. Consistent with this idea we found that all labeling was abolished by adding AMPA in the presence of NBQX (data not shown).

### Blocking endocytosis suppressed Fluoro-Gold Uptake

Since AMPA application is known to trigger clathrin-dependent receptor endocytosis [30] we hypothesized that this mechanism was responsible for the dye uptake. To test this idea, we examined the effects of bath-application of a hypertonic sucrose-ACSF solution (450mM), Dynasore, or a dynamin inhibitory peptide on the dye uptake. Hypertonic solutions have been shown to prevent the formation of coated-pits [31], thereby preventing receptor endocytosis; Dynasore has been shown to block dynamin and therefore internalization [32] and dynamin inhibitory peptide has also been shown to be a blocker of endocytosis [2, 33].

**Fig 6:**
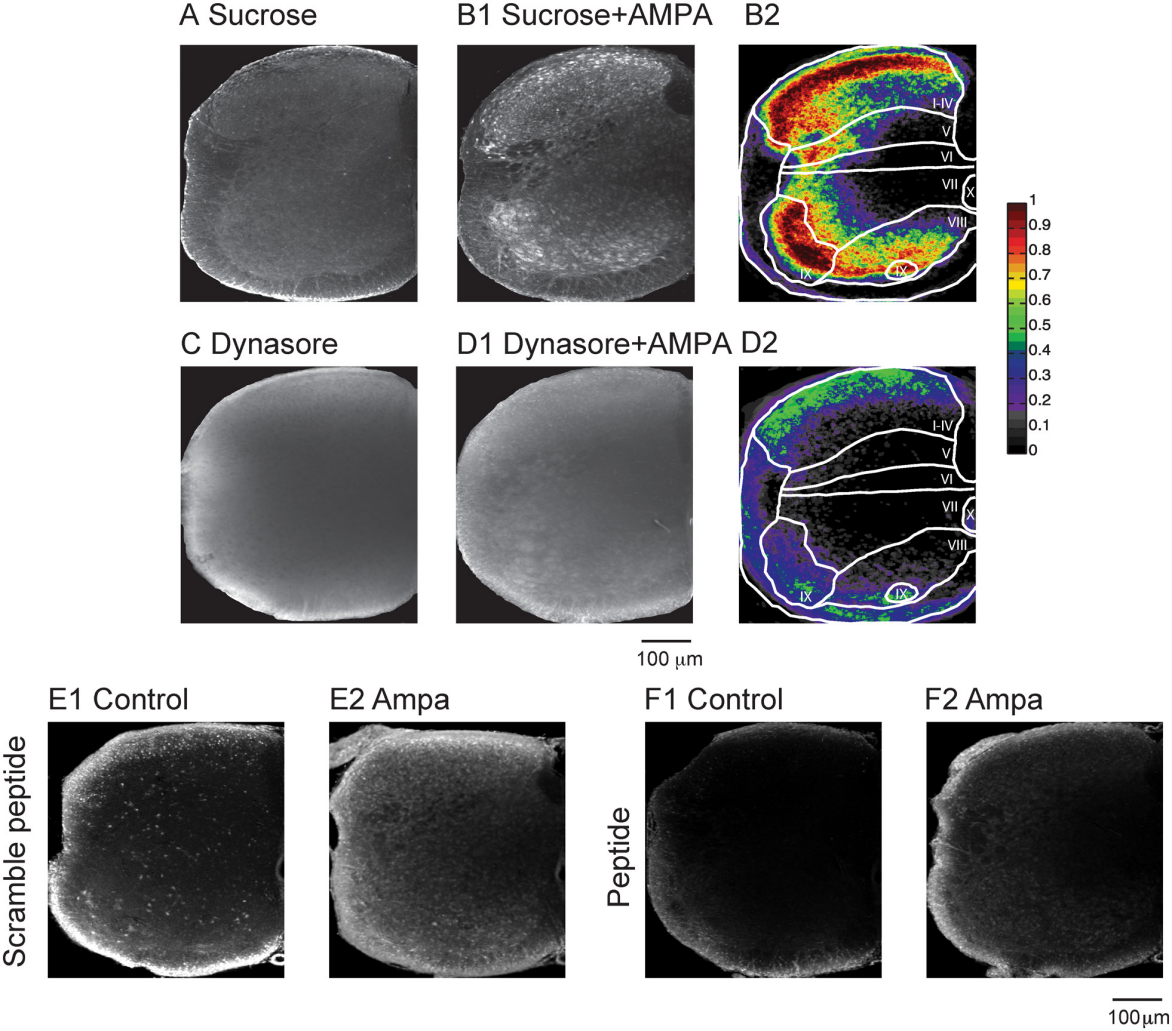
Fluoro-Gold uptake is abolished in control conditions, and is decreased when AMPA is applied in the presence of blockers of endocytosis. (A) Image of a hemicord when a hypertonic ACSF (450 mM sucrose) replaced the control ACSF. Neuronal labeling was almost abolished. (B1) Image of a hemicord when AMPA was applied together with hypertonic ACSF (5 μM). (B2) Probability map for AMPA and hypertonic ACSF. (C) Image of a hemicord when Dynasore (1 mM), a dynamin blocker, was applied (D1) When AMPA (5 μM) was co-applied with Dynasore cellular labeling was greatly reduced. (D2) Probability map for co-application of AMPA and Dynasore. The color bar to the right of panel B2 indicates the probability/pixel. (E1 –E2) Show FG uptake under control conditions and during AMPA application in the presence of the scrambled peptide. (F1–F2) Labeling is greatly reduced in the presence of the unscrambled dynamin inhibitory peptide.

We first established that application of a hypertonic solution does not irreversibly damage the cord by showing that locomotor activity could still be evoked by sacral root stimulation following washout of a hypertonic sucrose-ACSF solution (450mM; applied for 40 min. Data not shown). We then incubated three cords in a hypertonic sucrose-ACSF solution for 10 or 30 minutes prior to adding Fluoro-Gold. After this treatment, cell labeling was greatly reduced consistent with its mediation by endocytosis. For example, the labeling probability/pixel fell from the control value of 0.21 to 0.1 in laminae I-IV and from 0.32 to 0.15 in the lateral motor column, with similar reductions in all laminae (Fig 6A; Table 2). In presence of AMPA, the sucrose solution did not block labeling (Fig 6B), but it did decrease the probability of the labeling throughout the laminae compared to AMPA application alone (see Table 2).

When the dynamin blocker Dynasore (100 μM– 1mM) was applied, labeling was greatly reduced under resting conditions and in the presence of co-applied AMPA (Fig 6C and 6D). We also used the cell permeant myristoylated dynamin inhibitory peptide to block dynamin-dependent endocytosis when applied extracellularly. Bath–application of the inhibitory peptide (750 μM) reduced the labeling under control conditions and in the presence of AMPA (Fig 6F1 and 6F2). No such effects were observed using the scrambled version of the peptide (Fig 6E1 and 6E2). Collectively, these results are consistent with endocytosis as the uptake mechanism under control conditions and in the presence of glutamate agonists.

### Effect of AMPA and internalization on AMPA-induced currents recorded from spinal neurons

**Fig 7:**
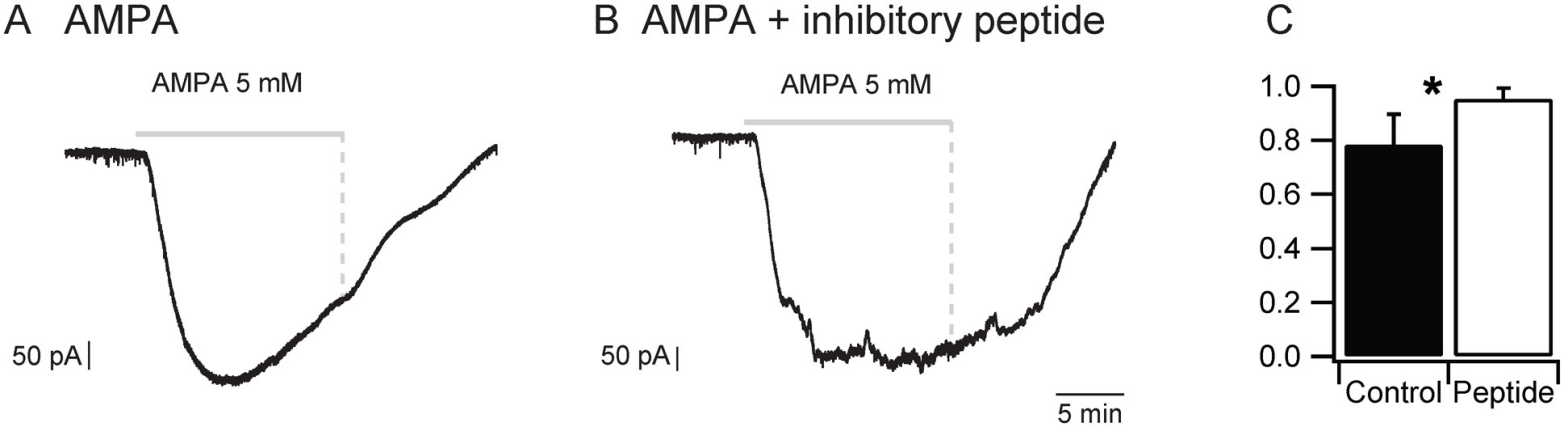
Dynamin inhibitory peptide prevents the decline of the AMPA-induced inward current. (A) Inward current induced in an L5 motoneuron during 15 min. application of AMPA administration under control conditions with no peptide in the electrode. Note the decline of the current in the presence of AMPA. (B) AMPA–induced inward current in the presence of dynamin inhibitory peptide in the patch electrode. (C) Quantification of the current decay (% maximum current in the last 1 min. of AMPA administration) under control conditions and in the presence of AMPA and the dynamin inhibitory peptide in the recording electrode.

To obtain additional evidence supporting endocytosis as the mechanism of Fluoro-Gold uptake we made whole cell recordings from spinal neurons during bath-application of AMPA. We examined the AMPA-evoked currents in spinal neurons using a dynamin inhibitory peptide inside the electrode to block endocytosis. These experiments were performed in the presence of TTX (1μM) and the inhibitory antagonists bicuculline (20μM) and strychnine (5μM) to block indirect synaptic effects. We bath applied AMPA (5μM) for 15 minutes and recorded the AMPA-evoked currents under voltage clamp (holding potential –70mV). Under control conditions (no inhibitory peptide), AMPA induced a large inward current that began to decay within 8.2 ± 3.5 min (n = 6) of AMPA administration (Fig 7A). However, in the presence of the dynamin inhibitory peptide, the current was maintained for the duration of AMPA application (Fig 7B). To quantify this difference, we measured the current amplitude over the last minute of AMPA application and expressed it as a percentage of the maximum current. We found that the current averaged over the last minute of the application fell to 78.3 ± SEM 0.05% of its peak value (n = 6, Fig 7A). By comparison, when the inhibitory peptide was included in the electrode, the current averaged over the last minute of AMPA application was 95.3 ± SEM 0.02% of the peak value (n = 5; Fig 7B). The difference in the averaged currents under the two conditions was statistically significant (t-test, p = 0.17). To eliminate any cholinergic contamination to the AMPA-induced current, we performed additional experiments to block cholinergic transmission (n = 3). For this purpose, we added nicotinic and muscarinic cholinergic antagonists (mecamylamine 50 μM; dhβE 50 μM; atropine 5 μM) to the TTX and inhibitory antagonists. To minimize complications arising from AMPA desensitization we added cyclothiazide (25 μM) to the aCSF. Furthermore, experiments in which the series resistance varied by more than 10% over the duration of the recording period were rejected. Under these conditions, we found that the AMPA-induced current averaged over the last minute of drug application declined to 82% of the peak current which was similar to the decline observed in the presence of TTX and inhibitory antagonists alone. This indicated that neither cholinergic nor desensitization effects complicated the experiments. Collectively, these results indicate the AMPA-induced current is due to a direct action on AMPA receptors and its decline is due to AMPA receptor internalization.

### Effects of the locomotor cocktail on Fluoro-Gold uptake

In the final set of experiments, we established if the drugs (serotonin and dopamine) that are frequently used to induce locomotor-like activity in the neonatal spinal cord also influenced AMPAR internalization. To establish if changes in AMPAR-internalization occur in the presence of these drugs, we bath-applied Dopamine (50μM) and Serotonin (10μM) individually and together in the presence of Fluoro-Gold. We found that both drugs affected Fluoro-Gold labeling. Dopamine resulted in enhanced labeling in the most dorsal part of laminae I-IV with little effect on the other laminae (Table 3). Serotonin similarly increased labeling in the dorsal part of laminae I-IV but reduced labeling in Laminae V-IX. When the drugs were added together labeling increased in lamiae I-IV.

**Fig 8:**
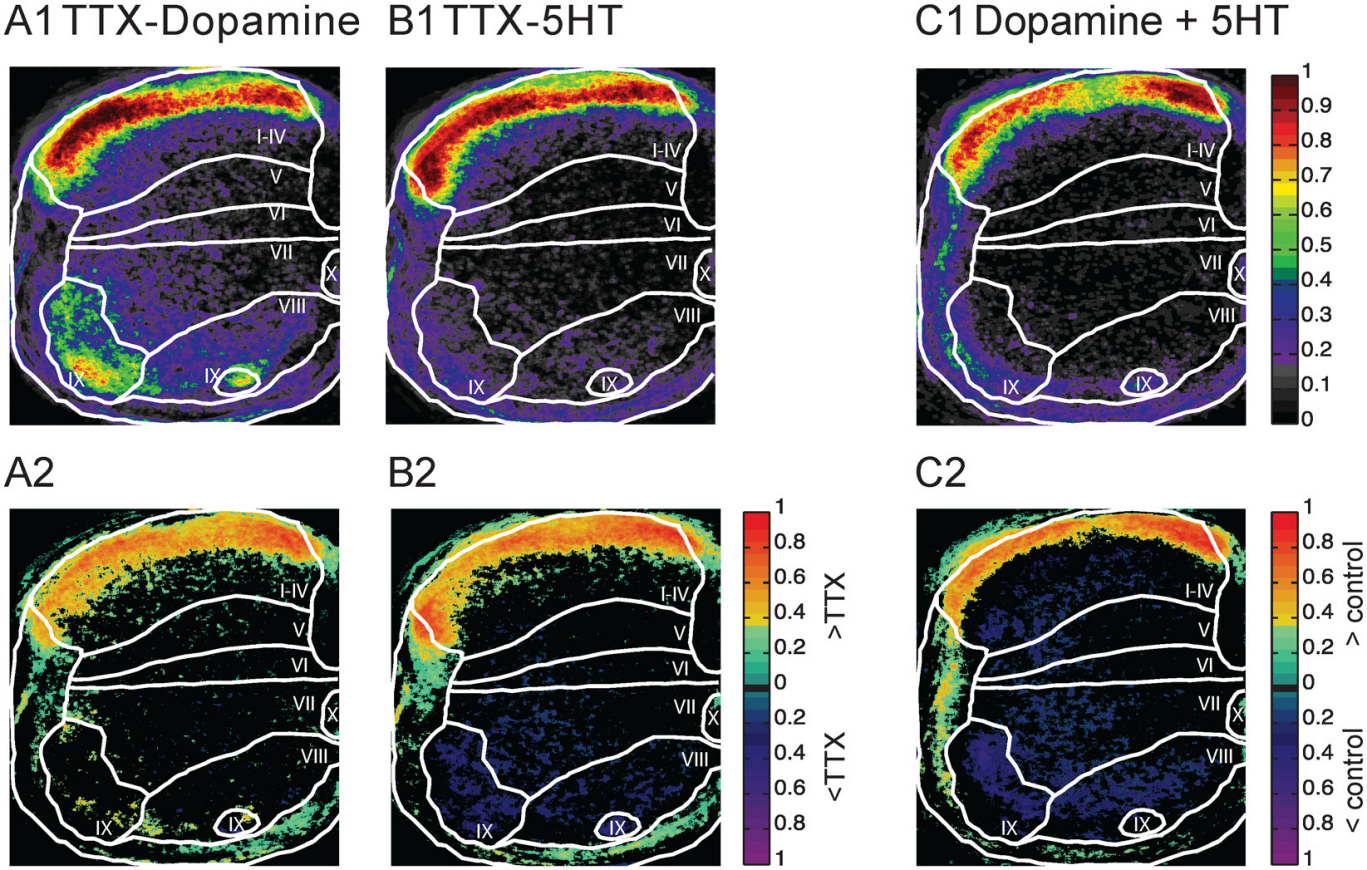
The effects of drugs used to initiate locomotor-like activity on Fluoro-Gold labeling in the presence of TTX. (A1–C1) Probability maps for Fluoro-Gold labeling in response to TTX and Dopamine (A1), TTX and Serotonin (5-HT). (B1) TTX, Dopamine, and 5-HT (C1). The color map to the right of A1, B1, and C1 shows the probability of labeling. (A2–C2) Difference maps compared to TTX for Fluoro-Gold labeling in response to TTX and Dopamine (A2), TTX and 5-HT (B2) and TTX and dopamine + 5-HT (C2). The colormap to the right of A2, B2, and C2 shows the color coding for pixels that differed statistically (p<0.05) from control.

Because the changes in Fluoro-Gold labeling might be caused in part by activation of the locomotor circuitry they may not reflect the action of the drugs alone. To overcome this concern, we repeated the experiments in the presence of TTX to block any neuronal activity induced by the drugs (Table 3 and Fig 8). We found that the drug-induced patterns of Fluoro-Gold labeling in the presence of TTX were very similar to those generated by the drugs alone. This result suggests that any activity induced by the locomotor drugs had little effect on the labeling pattern.

## Discussion

In this study, we have shown that AMPA-induced Fluoro-Gold labeling was greatly reduced by bath-application of the dynamin blocker Dynasore and the extracellularly applied, cell-permeant dynamin inhibitory peptide. In addition, AMPA-induced currents recorded in spinal neurons decayed spontaneously in the presence of the agonist, but were maintained when a dynamin inhibitory peptide was included in the electrode. To minimize complications due to retrograde labeling, the dye was administered for a brief time (thirty minutes) and the cut ventral roots were kept as long as possible. Retrograde labeling *in vitro* usually requires several hours to travel a few millimeters [34], as well as a dye concentration that is much higher (~100-200x) than that used in the present experiments. We conclude that extracellular Fluoro-Gold enters neurons together with AMPA receptors in response to AMPAR activation by dynamin-mediated endocytosis. The alternative explanation, that Fluoro-Gold enters the cell through the AMPA ion channel is not consistent with the abolition of labeling by inhibitors of endocytosis.

### Significance of endocytic uptake for the use of Fluoro-Gold as a retrograde and anterograde tracer

In the original paper describing the use of FG as a retrograde label hypothesized that the dye was taken up at synaptic terminals or damaged axons but not by fibers of passage [19, 28]. However, when the dye is injected into the vitreous humor of the eye it is taken up by retinal ganglion cells and transported anterogradely [28]. Thus, the dye must be taken up by the dendrites or cell bodies of retinal ganglion cells. We suggest that the mechanism of this uptake is likely to be transmitter-induced endocytosis.

Several papers have proposed that dye uptake occurs at synaptic terminals [16–18] which enables the dye to be used as an effective retrograde tracer. Indeed, this appears to be the mechanism responsible for the retrograde labeling of motoneurons following intraperioneal injection of the dye [16]. However, it has also been suggested that the dye can be taken up by undamaged fibers of passage [35]. This is a potential problem for retrograde labeling studies because the location of the synaptic terminals belonging to the labeled axons is unknown. Similarly, transmitter-induced uptake of Fluoro-Gold by undamaged cell bodies at the injection site will result in the same uncertainty concerning the location of the labeled cells’ synaptic terminals and will complicate the interpretation of retrograde loading experiments that assume the sole uptake mechanism involves synaptic terminals or damaged axons at the injection site.

### AMPAR-mediated internalization

Previous work has identified several forms of AMPAR internalization. Constitutive internalization occurs in the absence of externally applied ligands and affects AMPARs containing GluA1 or GluA2 subunits [36, 37]. We presume that this type of internalization accounts for the dye uptake we observe under control conditions. Most of the control labeling was abolished in the presence of AMPAR blockers, suggesting that it was mediated by extracellular glutamate.

Action-potential independent release of glutamate has been described in brainstem cranial visceral afferents that express TRPV1 (transient receptor potential vanilloid 1) channels which rapidly increased with temperatures > 25°C [38, 39]. Such receptors are present on afferents projecting into the spinal cord [40]. Although their constitutive activation could lead to glutamate release, this is likely to be low at the room temperature of our recordings. Nevertheless, there are other TRPV channels present on spinal neurons [41, 42] that might regulate asynchronous glutamate release. Consistent with this possibility, we found that bath-application of the broad spectrum TRP channel antagonist ruthenium red abolished all FG labeling (data not shown). However, the drug also blocked reflex and evoked neural activity so that we cannot be certain that its effects on loading were exclusively due to its action on TRP channels.

A second type of internalization occurs in response to ligand application and has been demonstrated in cultured hippocampal cells (for review see: [3, 20, 36, 43]) and in hippocampal slices [44]. In slices of the developing hippocampus, AMPA administration leads to a reduction in AMPAergic synaptic transmission presumably by AMPAR internalization [44]. Consistent with these reports, we found that AMPA or Kainate produced widespread FG labeling in all laminae of the cord and this labeling was blocked by the dynamin-inhibitor Dynasore or the extracellular dynamin inhibitory peptide. L-Glutamate and the excitatory amino acid transporter blocker TBOA produced a labeling pattern that was similar but weaker than that produced by AMPA or Kainate. It is possible that the less intense FG uptake produced by labeling with L-glutamate and TBOA is concentration related or alternatively, because the rate of internalization versus exocytosis is higher for Kainate and AMPA than for glutamate.

### Comparison with Cobalt labeling of Neurons

The labeling pattern we have described here has similarities to earlier work using cobalt to label neurons expressing calcium-permeable AMPA receptors [45, 46]. Cobalt is believed to enter the cell through calcium permeable ligand-gated AMPAR channels. In the dorsal horn, this was demonstrated by preventing cobalt labeling using Joro spider toxin which is believed to block cobalt entry at the channel pore [47]. Interestingly, the toxin did not block all of the cobalt labeling in response to kainate application suggesting that some other loading mechanism was involved. In addition, Nagy et al. [48] examined cobalt loading in the P14 rat spinal cord and reported cobalt loading for both AMPA and NMDA channels. Several reports, however, have failed to detect cobalt loading through NMDA channels [45, 47, 49, 50], presumably because the permeability of the NMDA channel to cobalt is very low [51]. It seems likely, therefore, that at least some of the loading observed in these earlier reports might be due to AMPAR-mediated endocytosis of cobalt. Consistent with this possibility, we found that FG loading may be facilitated by the presence of two positive charges on the molecule. We were unsuccessful in loading monovalent dyes (MEQ, AO), a monovalent anionic dye (Lucifer Yellow) or a neutral dye (FITC) either under control conditions or in the presence of AMPA. This observation raises the possibility that other divalent cations (calcium, magnesium, cobalt or more complex molecules) could gain access to the cytoplasm of neurons by AMPAR-mediated endocytosis.

### Relevance to drug-induced locomotion

Locomotor-like activity can be induced in the neonatal spinal cord by various mixtures of NMDA, dopamine and serotonin [23, 52]. We determined that these drugs all affected Fluoro-Gold labeling indicating that they can alter AMPAR trafficking. NMDA slightly enhanced the labeling observed under control conditions, consistent with previous reports demonstrating that AMPA receptor internalization can be mediated by NMDA receptor activation [53]. By contrast, serotonin and dopamine enhanced the labeling in the most superficial dorsal horn but suppressed labeling in all of the other laminae. These findings raise the possibility that the drugs may enhance network excitability by modulating the number and/or the composition of AMPA receptors on spinal neurons [54].

### Limitations of the technique for quantitative analysis of AMPA-mediated uptake in the intact spinal cord

One of the main limitations of the present work is that the diffusion time of FG was limited to 30 minutes and was not long enough to achieve a uniform concentration throughout the gray matter. This was particularly evident when AMPA was used to induce labeling and the central portion of the cord was more weakly labeled than anywhere else. That this was due to inadequate diffusion of FG to the central region of the cord was suggested by the uniform labeling seen when a hemicord was used. We chose the 30 min loading period to minimize the possibility of retrograde labeling along motor axons. We routinely used the whole cord instead of the hemicord, because of the possibility of retrograde labeling of the cut axons on the medial surface of the hemicord. The non-uniform distribution of the Fluoro-Gold concentration within the spinal cord precludes the use of Fluoro-Gold uptake for quantitatively studying AMPA-receptor endocytosis. However, as we have shown, the method can provide qualitative information about drug and lamina-specific AMPA-dependent endocytosis which is of interest to those studying network excitability and motor function. While the use of cultured neurons was outside the scope of the present work, Fluoro-Gold may be useful for quantitatively analyzing AMPA-mediated endocytosis in cultures where its concentration would be uniform. Moreover, the method could be validated by comparison with existing techniques for studying and quantifying endocytosis in culture.
